# Evaluating Plant Gene Models Using Machine Learning

**DOI:** 10.3390/plants11121619

**Published:** 2022-06-20

**Authors:** Shriprabha R. Upadhyaya, Philipp E. Bayer, Cassandria G. Tay Fernandez, Jakob Petereit, Jacqueline Batley, Mohammed Bennamoun, Farid Boussaid, David Edwards

**Affiliations:** 1 School of Biological Sciences, University of Western Australia, Perth, WA 6000, Australia; 22888361@student.uwa.edu.au (S.R.U.); philipp.bayer@uwa.edu.au (P.E.B.); cassandria.tayfernandez@research.uwa.edu.au (C.G.T.F.); jakob.petereit@uwa.edu.au (J.P.); jacqueline.batley@uwa.edu.au (J.B.); 2 Department of Computer Science and Software Engineering, University of Western Australia, Perth, WA 6000, Australia; mohammed.bennamoun@uwa.edu.au; 3 Department of Electrical, Electronic and Computer Engineering, University of Western Australia, Perth, WA 6000, Australia; farid.boussaid@uwa.edu.au

**Keywords:** gene models, pea, machine learning, XGBoost, SHAP

## Abstract

Gene models are regions of the genome that can be transcribed into RNA and translated to proteins, or belong to a class of non-coding RNA genes. The prediction of gene models is a complex process that can be unreliable, leading to false positive annotations. To help support the calling of confident conserved gene models and minimize false positives arising during gene model prediction we have developed Truegene, a machine learning approach to classify potential low confidence gene models using 14 gene and 41 protein-based characteristics. Amino acid and nucleotide sequence-based features were calculated for conserved (high confidence) and non-conserved (low confidence) annotated genes from the published *Pisum sativum* Cameor genome. These features were used to train eXtreme Gradient Boost (XGBoost) classifier models to predict whether a gene model is likely to be real. The optimized models demonstrated a prediction accuracy ranging from 87% to 90% and an F-1 score of 0.91–0.94. We used SHapley Additive exPlanations (SHAP) and feature importance plots to identify the features that contribute to the model predictions, and we show that protein and gene-based features can be used to build accurate models for gene prediction that have applications in supporting future gene annotation processes.

## 1. Introduction

The pace at which plant genomes are sequenced has increased significantly since the genome assembly of *Arabidopsis thaliana* in 2000, with many hundreds of plant genomes being sequenced, annotated, and published [1]. As more genomes are being sequenced, we have access to multiple assemblies of the same species, highlighting significant gene presence/absence variation and the need to construct pangenomes that reflect the gene content of a species rather than a single individual [2].

To understand a genome, it needs to be annotated with genes and other potentially functional units. Genome annotation is usually performed to identify coding genes and, where possible, assign a function to them. In contrast to the classic definition of a gene, a gene model can be defined as a region of the genome that can be transcribed into RNA that translates into a protein, or belongs to a class of non-coding RNAs [3,4]. Gene model prediction is a complex process in eukaryotes due to the presence of introns and splice variants, and is especially challenging in plants due to highly repetitive sequences, gene duplication, and transposable elements [5,6]. Two complementary approaches are usually applied for gene model prediction, homology based and ab initio methods. Homology based methods use sequence identity with known genes, or align expressed sequences such as RNA-Seq data to the assembly to help predict gene models. In contrast, ab initio methods identify structural elements of genes such as open reading frames (ORF) using statistical models, including Hidden Markov Models (HMM).

Although genome sequencing has become relatively straightforward, genome annotation remains a challenge, and the accuracy of gene prediction varies considerably. The statistical models used by ab initio annotation methods may require pre-existing, high-quality gene models, and the lack of pre-existing models decreases the accuracy of ab initio methods, therefore, genome annotation can be unreliable resulting in a significant number of false gene predictions [7]. To support studies relying on an accurate reference annotation and to avoid annotation errors propagating to other species, it is important to minimise genome annotation errors, such as failure to predict the presence of a real gene (false negative) or the prediction of a false positive gene call.

Genomes show conservation, structurally and functionally, between related species traced back to a common ancestor, and protein coding genes share sequence identity with homologous genes in other species. The presence of a large number of unique genes in a species would suggest the birth of novel genes following speciation, followed by their loss in subsequent speciation events. However, analysis of closely related species does not show evidence of a large number of novel genes, with most of the gene content conserved between closely related species [8,9,10,11]. This lack of evidence for the rapid birth of novel genes in species suggests that many of the predicted unique genes in a species may be false positive annotations and likely to be non-functional.

The application of machine learning in genome annotation can support more accurate gene annotation. Machine learning is a subcategory of artificial intelligence that sits at the crossover of computer science, statistics, and data science [12], and the ability of machine learning to build mathematical models and identify patterns in large datasets has been used for gene annotation. BALROG, a gene finding system for prokaryotes was developed by training a machine learning model on high quality prokaryotic genomes [13]. The functional elements in the human genome were identified using unsupervised machine learning [14]. Splice site prediction, an essential step in gene finding, has been performed using support vector machines [15]. A tree boosting scalable supervised machine learning algorithm eXtreme Gradient Boosting (XGBoost), has proven to be efficient in differentiating prophage genes from bacterial genes [16] and it was also used to predict gene loss in three *Brassica* species using genomic features [17].

Here, we present Truegene, a boosted tree-based solution to evaluate plant gene models based on protein and nucleotide attributes. Truegene is built to minimise false positive annotations and act as a support tool to provide more accurate structural annotation of genomes. We calculate both nucleotide and amino acid sequence features for each predicted gene. Truegene uses features including gene length, GC content, and codon adaptation index (CAI) to predict false positive gene models with an MCC score of 0.93 and an accuracy of 87%, making Truegene a valuable tool for the evaluation of gene models in plants.

## 2. Results

### 2.1. Feature Table Construction

The nucleotide and translated amino acid sequence datasets were derived from the annotated pea genome [18]. A total of 34,427 genes that shared sequence identity with other species in the NCBI NR database were considered high confidence conserved genes, while 10,793 genes that were not found in the NCBI NR database were considered low confidence non-conserved genes. Two feature tables were constructed by calculating 41 features for the amino acid sequences and 14 features for the nucleotide sequences (Supplementary Tables S1 and S2). We conducted Pearson correlation tests to investigate which features are correlated (Table 1 and Table 2).

### 2.2. Performance Evaluation

**Figure 1:**
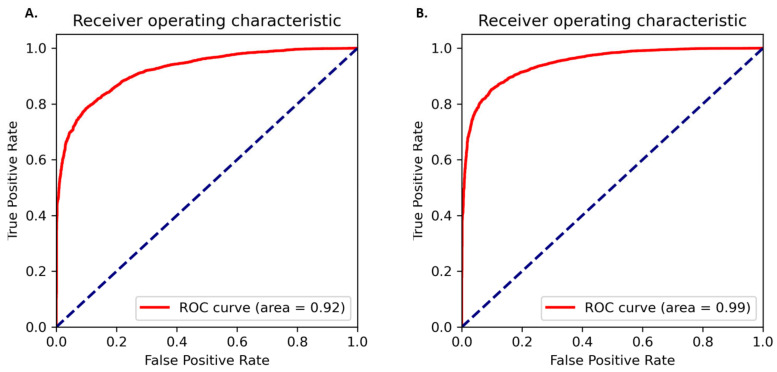
AUROC curves for the (**A**) protein model and (**B**) nucleotide model. The true positive rate is plotted against false positive rate at different classification thresholds.

**Figure 2:**
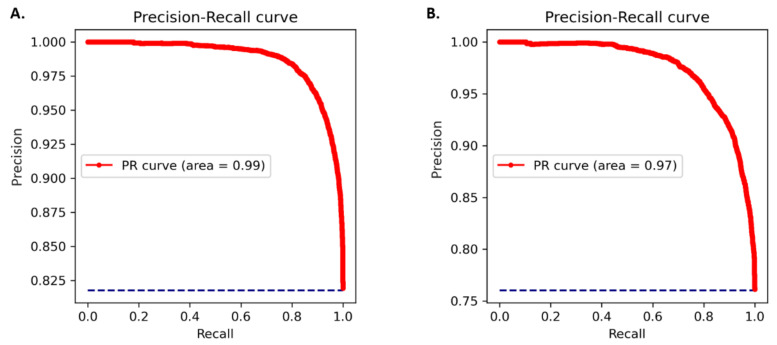
PR curves for (**A**) protein model (**B**) nucleotide model. Precision is plotted against recall at different probability thresholds.

**Figure 3:**
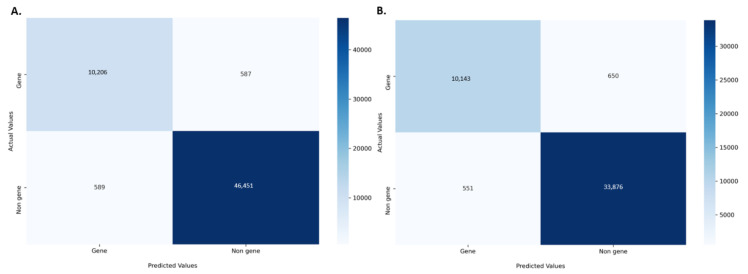
Confusion matrix for the (**A**) protein model (**B**) nucleotide model. The matrix is coloured based on the number of sequences in each class.

The amino acid and nucleotide models were fine-tuned, and the optimised models were tested for prediction accuracy. The prediction performance of the model on the test dataset was calculated by comparing the result with the expected values. The optimized protein model classifier had a slightly better prediction accuracy than the nucleotide model (89.92% compared to 86.72%). We evaluated the models using 10-fold cross validation, Area Under Receiver Operator Curve (AUROC), Precision Recall (PR) curve, F-score (F1), Mathew’s correlation coefficient (MCC), and confusion matrix. The high prediction accuracy for the models is supported by the evaluation metrics (Table 3). Cross validation showed the protein model to have a slightly higher testing accuracy, 88.66 ± 0.65% compared to 85.38 ± 0.40%, however, the MCC score for both the models was 0.93 indicating accurate predictions. The AUROC curve demonstrates that the model can distinguish the classes (conserved or non-conserved) (Figure 1), while a high area under the PR curve demonstrates a low false positive rate and low false negative rate (Figure 2). From the confusion matrix it can be observed that the number of misclassified classes (false positive and false negative) is minimal (Figure 3).

### 2.3. Model Explanations

**Figure 4:**
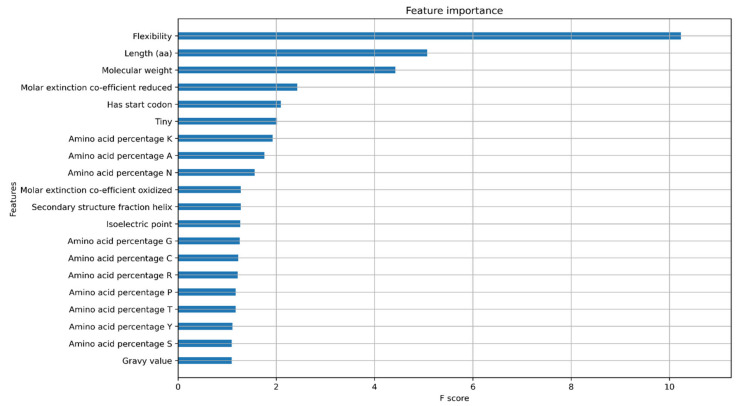
Feature importance Gain plot for XGBoost protein classifier model showing the top 20 features contributing to the model.

**Figure 5:**
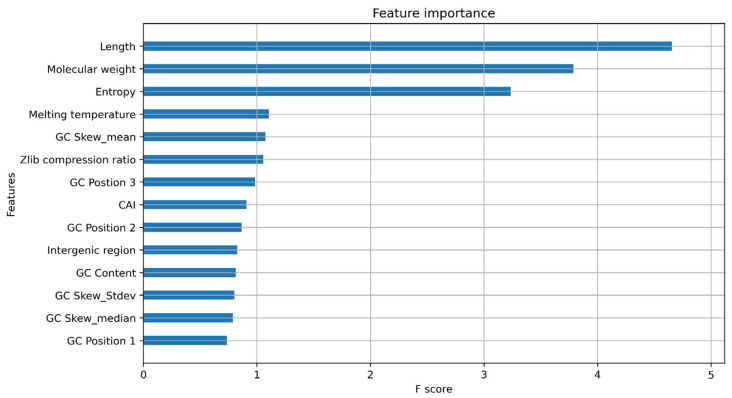
Feature importance gain plot for nucleotide model showing the 14 features contributing the model. CAI = Codon Adaptation Index, GC_Stdev: Standard deviation of GC skew value.

A key component of machine learning is explainable AI, which involves understanding the features that are important for model prediction. A common way to look at model explanations is feature attribution, where a score is assigned to each feature associated with its contribution to the prediction. We assessed the feature importance for both models using XGBoost feature importance plots and SHapley Additive exPlanations (SHAP) values. The XGBoost feature importance plots are used to interpret the relative importance of each feature to the model and the number of observations for each feature (Supplementary Figures S1 and S2). The XGBoost gain plot shows how valuable each feature is for the construction of the boosted trees within the model and the improvement it brings to the accuracy of the model (Figure 4 and Figure 5).

**Figure 6:**
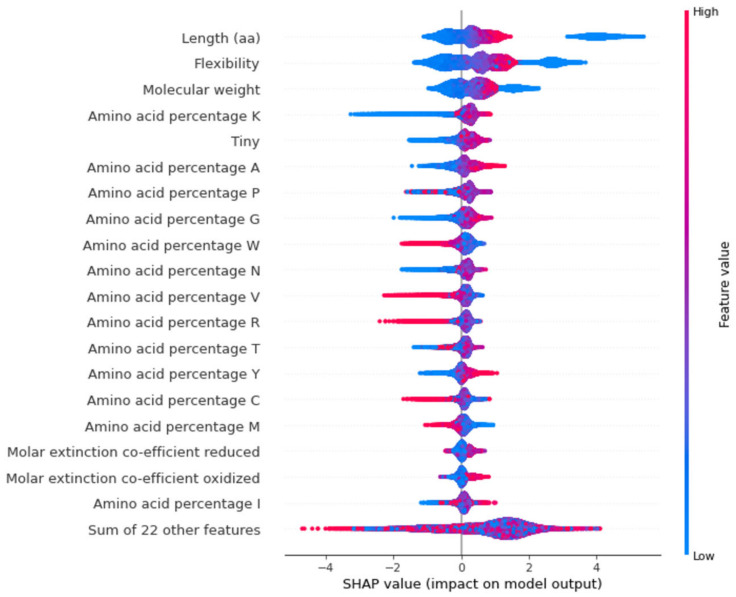
Beeswarm plot for top 20 features that contribute to protein model. Each dot indicates one value, and they pile up in each row to show density. The red dots represent higher feature value while the blue dots represent lower feature value. The positive side indicates high confidence genes while the negative side indicates low confidence genes.

**Figure 7:**
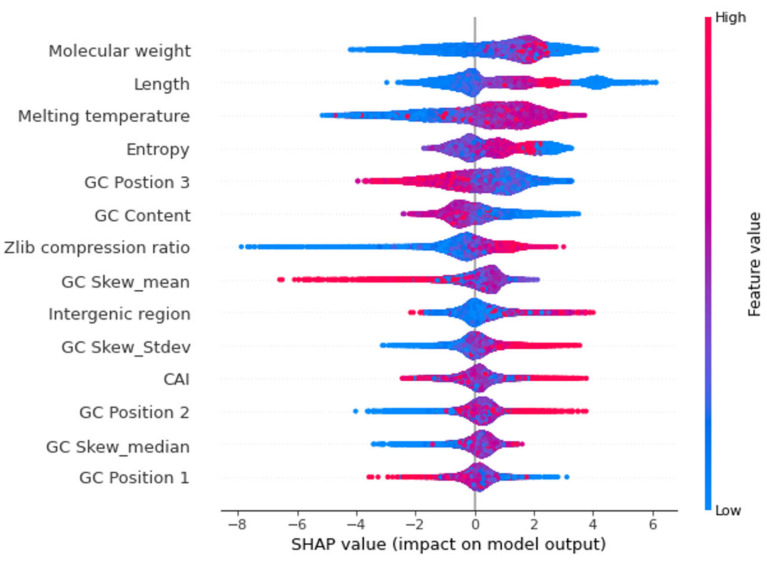
Beeswarm plot for the 14 features that contribute to nucleotide model. Each dot indicates one value, and they pile up in each row to show density. The red dots represent higher feature value while the blue dots represent lower feature value. The positive side indicates high confidence genes and while the negative side indicates low confidence genes.

SHAP values can provide a clear assessment of the feature’s importance and their interaction with the predictions. SHAP not only illustrates the important features but also shows how the features influence the prediction (positive or negative) [19,20]. The contribution of each feature to the output can be identified and therefore the relationship between the biological significance and feature importance to the model can be established. The beeswarm summary plots show the relationship of the feature to the target variable (Figure 6 and Figure 7). Length and molecular weight were observed to have the strongest relationship with the target variable in both models. In addition, the protein model exhibits flexibility and the presence of a canonical start codon as the top features, with the melting temperature and GC content being the top features for the nucleotide model.

## 3. Discussion

In this study, we successfully built and optimised gene classifier models with accuracy scores ranging between 87–90%. The protein model had a slightly higher prediction accuracy compared to the nucleotide model. However, MCC scores for both models were similar indicating equivalent prediction quality. MCC has considered a reliable metric as the MCC score is only high when the model’s prediction produced good results in all four components of the confusion matrix [21].

In the protein model, the most distinguishing feature between classes is the protein sequence length, which positively correlates with the related features of flexibility and molecular weight. However, the higher F-score for flexibility suggests that flexibility contributes directly to the model (Figure 4). Prior studies have shown that flexibility allows the protein to interact with other molecules making it an important feature [22]. As longer proteins were seen to be clustered on the positive side of the SHAP plot while shorter proteins were distributed on both sides, the protein length has a bimodal distribution and cannot be used as a single feature for confidence prediction (Figure 6).

The presence of a canonical start codon was also observed as a major feature (Figure 4). The presence of a translation initiation codon has been used previously by gene finding software [23]. Any protein coding gene is translated only when the ribosomes encounter the start codon. Therefore, the model identifies the presence of methionine at the start of the gene as an important feature. However, the model only reads methionine as a start codon, and some real genes have no traditional start codon, with translation initiation using a non-AUG start codon [24]. Non-AUG codons are not always transcribed to methionine, with CUG, transcribed into leucine, also acting as start codon [25,26]. This is a potential limitation of the model that may be addressed by using larger datasets and validated genes that have non-AUG start codons.

Gene length, molecular weight, entropy, and melting temperature were found to be the most important features of the nucleotide model (Figure 7). This is consistent with a study that uses gene features to distinguish between genomes [16]. As with the protein model, length showed a high positive correlation with molecular weight (R = 0.99) and Shannon entropy (R = 0.83), indicating that these values were related. Sequence length has previously been shown to be a distinguishing character for gene prediction [16,27]. While studies show that false positive gene calls tend to be shorter [28], not all small genes are associated with annotation errors as some small genes play important roles in cellular function, signalling, and enzymatic activity [29]. Therefore, gene sequence length is bimodal with shorter genes being ambiguous and so this cannot be used as a single distinguishing feature.

We observed that GC content was one of the important features used by the model to discriminate between confident and non-confident gene models, with genes having lower GC content on the positive side of the SHAP plot, highlighting that these are more likely to be predicted as real genes (Figure 7). GC content has been used as a feature by various annotation programs [30,31]. GeneMark.hmm, a gene prediction program, uses GC content as an important feature to estimate gene models [32]. The average GC content for the pea genome was calculated to be 37.61% with the average GC content of the annotated genes at 42.39%.

Since the melting temperature is related to the stability of the DNA structure [33] with GC rich sequences having a higher melting temperature than AT rich sequences, we observed a positive correlation between GC content and melting temperature. Here, a high melting temperature for a gene contributes towards a positive prediction, that it is a confident gene (Figure 7). While this seems to conflict with the observation that genes with a lower GC content are likely to be confident genes, the correlation of GC content and melting temperature of high confidence genes is less than low confidence genes (0.97 compared to 0.89). A heatmap of GC content versus melting temperature shows that the model classifies genes with a GC content of around 40% and melting temperature of around 80° as confident genes (Supplementary Figures S3 and S4). The average GC content for high confidence genes was calculated to be 42.12% which is lower than low confidence genes (43.25%), indicating the model recognises genes with lower GC as high confidence genes. Previous studies have also shown that melting temperatures are higher at transcription sites in mouse and human genes [34]. In *Oryza sativa*, there was a clear difference in the melting temperature profile between gene introns and exons, and is consistent with our model identifying melting temperature as an important feature in predicting confident pea genes.

The Zlib compression ratio is a data compression method that is related to the number of repeats in DNA sequence data and is calculated as the ratio of data size after compression compared to before compression [35]. A ratio of 1 indicates no compression, denoting few if any repeats, while a lower ratio indicates the presence of repetitive sequences. Genes with higher Zlib ratios are present on the positive side of the SHAP plot suggesting that genes with less repetitive sequences are more likely to be real genes (Figure 7). This reflects the observation that repetitive sequences are often associated with non-coding regions of the genome.

## 4. Materials and Methods

### 4.1. Dataset

The *Pisum sativum* Cameor annotated gene and protein dataset was obtained from the Legume Information System (LIS) [36]. The annotated genes were compared with the NCBI NR database, and the gene models that failed to show sequence identity (excluding pea) with an e-value lower than 0.01 were considered low confidence non-conserved gene models, resulting in 34,427 high confidence genes and 10,793 low confidence genes. Amino acid and nucleotide features were selected based on Sirén et al. [16].

### 4.2. Model Training

Tables representing 41 amino acid and 14 nucleotide acid features were generated using Python (v3.8) and Biopython packages [37] (Supplementary Table S1). Pearson correlation tests were performed using the Python SciPy stats package (v1.6.2) [38]. The Pandas package (v1.2.5) [39] was used to examine and employ the feature table data and the Scikit-learn package (v0.22.1) [38] was used to split the data into training and test datasets using the train_test_split function. The dataset was split into 80% training data and 20% testing data. An initial XGboost model was built with default parameters. An optimal parameter search was performed using Python package Scikit-Optimize (v.0.8.1) via Bayesian Optimization (BayesSearchCV). The model hyperparameters were fine-tuned using the following settings: learning_rate: 0.01–1.0, min_child_weight: 0–10, max_depth: 0–50, max_delta_step: 0–20, subsample: 0.01–1.0, colsample_bytree: 0.01–1.0, colsample_bylevel: 0.01–1.0, reg_lambda: 1 × 10^−9^–1000, reg_alpha: 1 × 10^−9^–1.0, gamma: 1 × 10^−9^–0.5, min_child_weight: 0–5, n_estimators: 50–200, scale_pos_weight: 1 × 10^−6^–500.

XGBoost (v.1.3.3) [40] was used to construct two XGBClassifier models using the optimised parameters (Supplementary Table S2). The XGBClassifier was fitted using the training dataset and the two XGBoostClassifier models were constructed with the goal to predict whether a given gene or protein is likely to be a low confidence annotation. The model’s performance was then validated by running it on the 20% testing data, and the model prediction accuracy was calculated using the accuracy_score function. The model is implemented in a Jupyter notebook hosted at GitHub: https://github.com/AppliedBioinformatics/Truegene (accessed on 16 May 2022).

### 4.3. Model Evaluation

A series of evaluation metrics were calculated using the scikit-learn library, Sklearn.metrics package (v0.22.1) [38] to test the accuracy of the model. 10-fold cross validation, AUROC, PR curve, F1, MCC, and confusion matrix were used as the evaluation metrics. The AUROC curve is obtained by plotting the true positive rate against the false positive rate at different classification thresholds. F1 and MCC were calculated according to the standard formula [21]. 

The in-built XGBoost feature importance functionality was used to determine and plot feature importance. The plots were visualized using matplotlib (v.3.1.1) [41], and explained using SHapley Additive exPlanations (SHAP). To understand the contribution of each feature for training and testing, the Shapley explanation values were plotted as SHAP plots (v.0.39.0) [20].

## 5. Conclusions

Our model can accurately differentiate between high and low confidence genes based on a range of protein and nucleotide features. The most significant features of the model are the length of the gene, presence of start codon, melting temperature of the gene, and GC content. While this model is based on pea genes, it can be extended by including other species and analysing additional, more complex features such as predicted protein structures. Truegene supports automated gene model evaluation in plant genomes as it can identify low confidence predictions with high accuracy. With the increasing number of plant genomes being sequenced and the need for accurate gene annotation, approaches such as Truegene can provide additional quality control. This computational method determines with 87–90% accuracy whether a gene is conserved among species and therefore is likely to be a true gene, and while it cannot determine with certainty whether a gene model is correct, it can identify gene models that should be examined in more detail for further validation. Our study resulted in a reusable classifier model that can act as a support tool to validate the gene models and improve gene prediction.

## Figures and Tables

**Table 1:** Pearson correlation coefficient for protein features with *p*-value < 0.05.

Feature 1	Feature 2	Correlation Co-Efficient (R)
Length	Flexibility	0.99
Length	Molecular weight	0.99
Length	Molar extinction coefficient reduced	0.81
Length	Molar extinction coefficient oxidised	0.81
Aliphaticity	Aliphatic index	0.93
Gravy Value	Non-polar amino acids	0.86
Tiny amino acids	Amino acid percentage G	0.57
Iso-electric point	Acidic amino acids	−0.58
Tiny amino acids	Amino acid percentage A	0.48

**Table 2:** Pearson correlation test for nucleotide features with *p*-value < 0.05.

Feature 1	Feature 2	Correlation Co-Efficient (R)
Length	Molecular weight	0.99
Length	Entropy	0.83
Length	Melting temperature	0.17
Length	Zlib compression ratio	−0.68
GC content	Melting temperature	0.92
GC content	GC at position 3	0.72
GC content	GC at position 2	0.71
GC content	GC at position 1	0.58
Molecular weight	Entropy	0.83

**Table 3:** Evaluation metrics for XGBoost classifier models.

Evaluation Metric	Protein Model	Nucleotide Model
Prediction Accuracy	89.92%	86.72%
10-fold cross validation	88.66% (±0.65%)	85.38% (±0.40%)
F1_score	0.94	0.91
Average precision score	0.93	0.90
MCC	0.93	0.93
AUC value	0.94	0.92

## Data Availability

The *Pisum sativum* Cameor annotated gene and protein datasets are available at Legume Information System database [36]. Available online: https://v1.legumefederation.org/data/public/Pisum_sativum/Cameor.gnm1.ann1.7SZR/ (accessed on 26 August 2020). Truegene model source code used in this study is openly available at: https://github.com/AppliedBioinformatics/Truegene (accessed on 2 May 2022).

## Supplementary Materials

The following supporting information can be downloaded at: https://www.mdpi.com/article/10.3390/plants11121619/s1. Table S1. List of protein features calculated for the high confidence and low confidence amino acid sequences; Table S2. List of nucleotide features calculated for the high confidence and low confidence nucleotide sequences. Figure S1. XGBoost Cover plot for (A) Protein model shows tops 20 features (B) Nucleotide model. CAI = Codon Adaptation Index, GC_Stdev: Standard deviation of GC skew value; Figure S2. XGBoost Weight plot for (A) Protein model shows top 20 features (B) Nucleotide model. CAI = Codon Adaptation Index, GC_Stdev: Standard deviation of GC skew value; Figure S3. Correlation between GC content and Melting temperature. True positive refers to the high confidence conserved genes and False positive refers to low confidence non-conserved genes; Figure S4. Heatmap of GC content plotted against melting temperature.
