# Supplementary material for: Evaluating Plant Gene Models Using Machine Learning
Source: Plants (Basel). 2022 Jun 20;11(12):1619. doi: 10.3390/plants11121619 (PMC9230120; doi:10.3390/plants11121619)
Supplement: Supplementary file 1 [file plants-11-01619-s001.zip › plants-1751862-supplementary.pdf]

Supplementary Materials

Evaluating Plant Gene Models Using Machine Learning

Shriprabha R. Upadhyaya [1], Philipp E. Bayer [1], Cassandria G. Tay Fernandez [1], Jakob Petereit [1], Jacqueline Batley [1], Mohammed Bennamoun [2], Farid Boussaid [3] and David Edwards [1,*]

[1] School of Biological Sciences, University of Western Australia, Perth, WA 6000, Australia; 22888361@student.uwa.edu.au (S.R.U.); philipp.bayer@uwa.edu.au (P.E.B.); cassandria.tayfernandez@research.uwa.edu.au (C.G.T.F.); jakob.petereit@uwa.edu.au (J.P.); jacqueline.batley@uwa.edu.au (J.B.)

[2] Department of Computer Science and Software Engineering, University of Western Australia, Perth, WA 6000, Australia; mohammed.bennamoun@uwa.edu.au (M.B.);

[3] Department of Electrical, Electronic and Computer Engineering, University of Western Australia, Perth, WA 6000, Australia; farid.boussaid@uwa.edu.au (F.B.)

* Correspondence: dave.edwards@uwa.edu.au

Table S1. List of protein features calculated for the high confidence and low confidence amino acid sequences.

Protein Feature name	Number of features	Module used
Amino acids sequence length	1	Biopython Prot param
Start codon	1	Biopython Prot param
Amino acid percentage	20 – all amino acids	Biopython Prot param and Alphabet
Isoelectric point	1	Biopython Prot param
Grand Average of Hydropathy (GRAVY) value	1	Biopython Prot param
Molecular weight	1	Biopython Prot param
Instability Index	1	Biopython Prot param
Secondary structure fraction	3 – helix, turn, sheet	Biopython Prot param
Flexibility	1	Biopython Prot param
Aromaticity	1	Biopython Prot param
Molar extinction co-efficient	2 – oxidised, reduced	Biopython Prot param
Aliphatic index	1	In-house script
Aliphaticity	1	In-house script
Charge	1	In-house script
Polar amino acids	1	In-house script
Nonpolar amino acids	1	In-house script
Acidic amino acids	1	In-house script
Basic amino acids	1	In-house script
Tiny amino acids	1	In-house script

Table S2. List of nucleotide features calculated for the high confidence and low confidence nucleotide sequences.

Nucleotide feature name	Number of features	Module used
Nucleotide sequence length	1	Biopython SeqUtils
GC Content	1	Biopython SeqUtils
GC position	3 – 1st, 2nd and 3rd	Biopython SeqUtils
GC skew	3 – Mean, Median, Standard deviation	Biopython SeqUtils
Melting temperature	1	Biopython SeqUtils
Molecular weight	1	Biopython SeqUtils
Shannon entropy	1	Scipy stats
Zlib compression ratio	1	Python zlib, in-house script
Codon Adaptation Index (CAI)	1	Biopython Alphabet, in house script
Intergenic region	1	Python, in house script

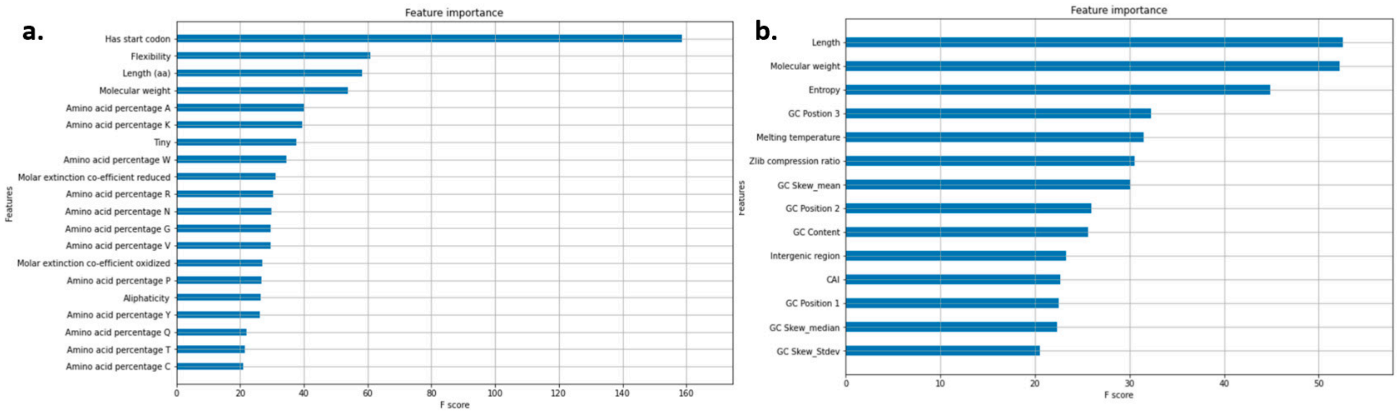

Figure S1. XGBoost Cover plot for (**a**) Protein model shows tops 20 features (**b**) Nucleotide model. CAI = Codon Adaptation Index, GC_Stdev: Standard deviation of GC skew value.

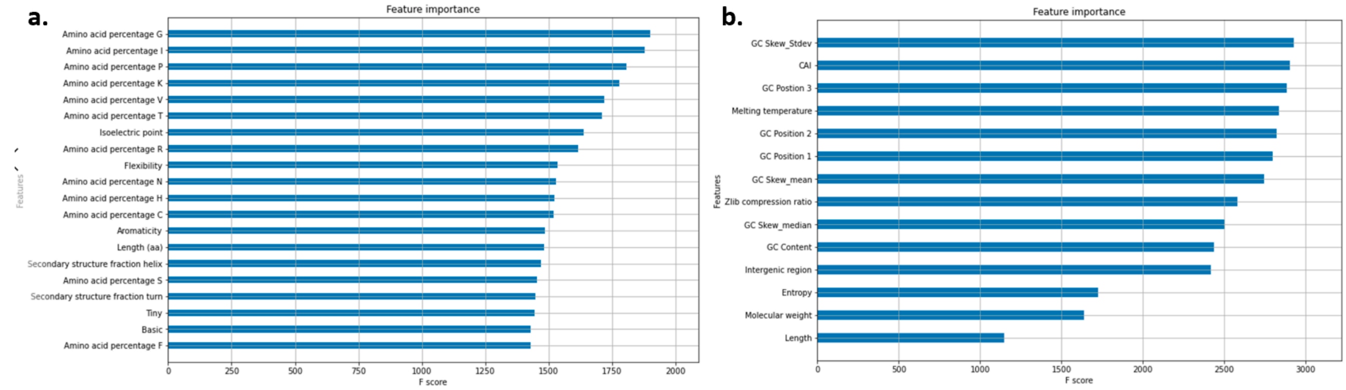

Figure S2. XGBoost Weight plot for (**a**) Protein model shows top 20 features (**b**) Nucleotide model. CAI = Codon Adaptation Index, GC_Stdev: Standard deviation of GC skew value.

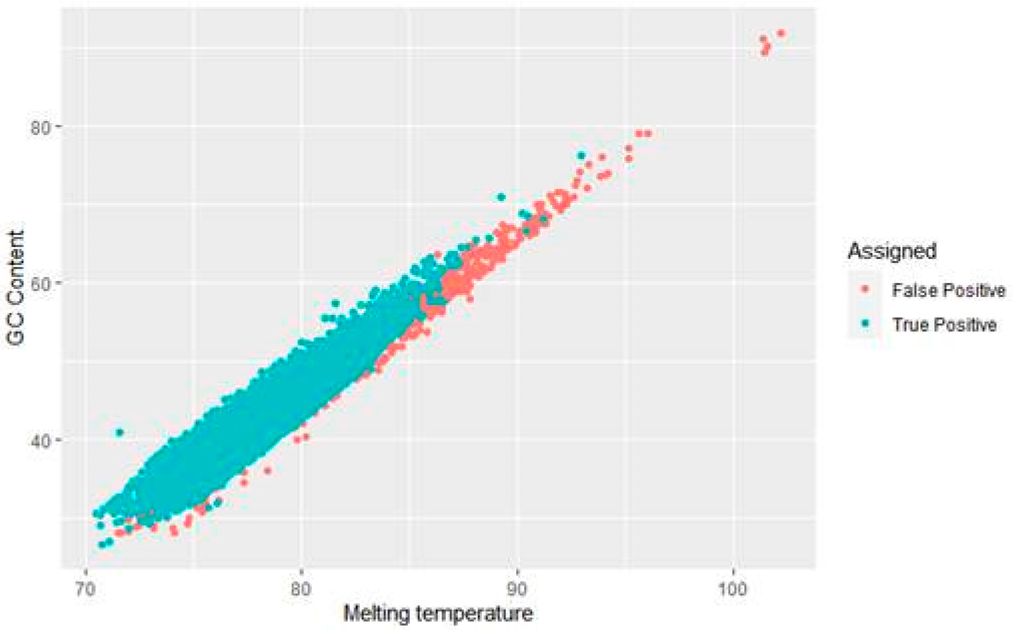

Figure S3. Correlation between GC content and Melting temperature. True positive refers to the high confidence conserved genes and False positive refers to low confidence non-conserved genes.

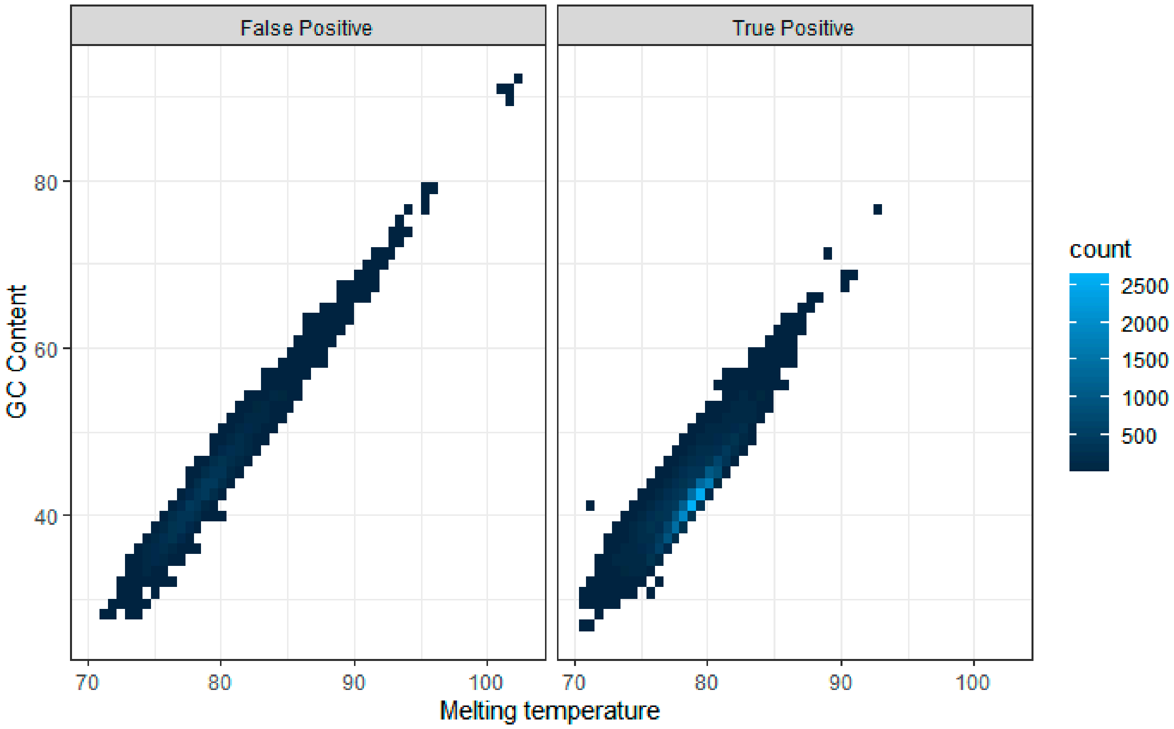

Figure S4. Heatmap of GC content plotted against melting temperature.
